# Medicine in words and numbers: a cross-sectional survey comparing probability assessment scales

**DOI:** 10.1186/1472-6947-7-13

**Published:** 2007-06-11

**Authors:** Cilia LM Witteman, Silja Renooij, Pieter Koele

**Affiliations:** 1 Diagnostic Decision Making, Behavioural Science Institute, Faculty of Social Sciences, Radboud University Nijmegen, P.O. Box 9104, 6500 HE Nijmegen, The Netherlands; 2 Decision Support Systems, Department of Information and Computing Sciences, Faculty of Science, Utrecht University, P.O. Box 80089, 3508 TB Utrecht, The Netherlands; 3 Methodology Section, Department of Psychology, University of Amsterdam, Roetersstraat 15, 1018 WB Amsterdam, The Netherlands

## Abstract

**Background:**

In the complex domain of medical decision making, reasoning under uncertainty can benefit from supporting tools. Automated decision support tools often build upon mathematical models, such as Bayesian networks. These networks require probabilities which often have to be assessed by experts in the domain of application. Probability response scales can be used to support the assessment process. We compare assessments obtained with different types of response scale.

**Methods:**

General practitioners (GPs) gave assessments on and preferences for three different probability response scales: a numerical scale, a scale with only verbal labels, and a combined verbal-numerical scale we had designed ourselves. Standard analyses of variance were performed.

**Results:**

No differences in assessments over the three response scales were found. Preferences for type of scale differed: the less experienced GPs preferred the verbal scale, the most experienced preferred the numerical scale, with the groups in between having a preference for the combined verbal-numerical scale.

**Conclusion:**

We conclude that all three response scales are equally suitable for supporting probability assessment. The combined verbal-numerical scale is a good choice for aiding the process, since it offers numerical labels to those who prefer numbers and verbal labels to those who prefer words, and accommodates both more and less experienced professionals.

## Background

Reasoning under uncertainty is common practice in the medical field. Diagnoses and prognoses are always made in the face of uncertainty, for example about the exact pathogenic processes underlying some observed relation between symptom and disease. In addition, most diagnostic tests are not 100% reliable, resulting in uncertainty as to the true presence or absence of the disease tested for. On top of that, the effects of treatment may differ per patient and cannot be predicted with certainty. Clinical decision making is, in short, a complex task which could benefit from supporting tools.

Support may for example be provided by the increasingly recommended 'threshold approach' [1]. This approach defines two thresholds. The first threshold indicates the decision boundary between no treatment and testing. If the clinician's estimate of the probability of the presence of a disease falls below this threshold, no treatment is given. The second threshold is the boundary between testing and treating. Probability estimates of the presence of a disease which fall between these two thresholds dictate performing additional diagnostic tests, and estimates which are above the second threshold indicate that treatment should be started right away. The approach is only valid if the physician's estimate is a true probability, reasonably accurate and unbiased. It is well-known, however, that humans are poor probability estimators [2] and that physicians are no exception [3,4]. This observation can be partially explained by 'support theory' [5], which is based on the idea that subjective probabilities are not true probabilities of events in a mathematical sense, but that they reflect how much support people have for different descriptions of the events. As a result, subjective probability estimates have been shown to suffer from unpacking effects and subadditivity. The typical unpacking effect refers to the phenomenon that when an hypothesis is unpacked into a number of more detailed hypotheses, then the sum of the probabilities assigned to the more detailed descriptions exceeds the probability estimated for the 'packed' hypothesis. When the sum of the probabilities assigned to all hypotheses exceeds a 100%, this overestimation of the true probabilities is called subadditivity. It has been shown that probability estimates provided by physicians are also prone to the unpacking effect [6,7], which "questions the applicability of the threshold approach if the physicians are not given guidance, explicit tools and formal training in probability estimation" ([7], p. 763).

**Figure 1:**
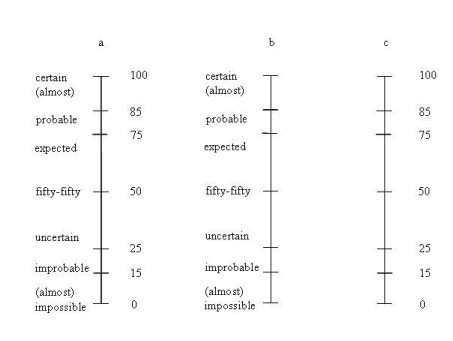
The probability scales: The verbal-numerical (a), the verbal (b) and the numerical (c) probability scale.

Different methods are available to support experts in assessing probability judgments (for an overview see e.g. [8-10]). However, in constructing a Bayesian network as part of a decision support system to aid physicians in selecting a suitable treatment for patients with oesophageal cancer, we found that none of the standard probability elicitation methods seemed to work (cf. below: Context). We therefore designed our own method [11]. A major ingredient of this method is the use of one probability scale with both verbal and numerical labels (see Figure 1a below), which is similar to but differs from methods proposed by others (e.g. [12]), who provide both a verbal and a numerical scale but separately. The method, including the double scale, has since been used for eliciting the required probabilities in a number of realistic applications of decision support systems, both in the medical field [11,13,14] as well as in other domains [15,16], yet a more extensive validation of the scale is still called for. In a previous between-subject study, we evaluated our verbal-numerical probability scale with students as subjects, comparing the ease of use of and accuracy of assessments on our verbal-numerical scale against a purely numerical scale [17]. In the study reported in the current paper, we had physicians as subjects (general practitioners (GPs)), and we included a scale with only verbal labels. We were interested in: their preferences in use of the three scales, differences in the assessments, confidence in assessments with the three scales, and whether or not the use of a response scale affected the unpacking effect described above.

### History of the verbal-numerical probability scale

Our personal experiences with eliciting probabilities using our verbal-numerical probability scale were very good [11]. However, anyone who considers using our double scale, should be familiar with its underlying ideas. For this reason we review its design, initial use and first evaluation.

#### Context

The verbal-numerical probability scale was designed as part of a probability elicitation method for the fast assessment of 4000 point probabilities required for the construction of a real-life Bayesian network in the domain of oesophageal cancer (for details on the network and its construction we refer to [11]). Bayesian networks are mathematical models that capture a joint probability distribution over a set of variables that are relevant in the domain of application [18], and are popular in the medical domain (see e.g. [19-22]). Bayesian networks allow for the computation of any prior or posterior probability of interest, using efficient algorithms that basically implement Bayes' rule.

The numerical probabilities required for a network's specification can be easily established from a very large, rich and reliable data set. However, such data sets are rare and often do not allow the reliable assessment of the typically large number of specific probabilities that are required. As a result, some or all of the probabilities will have to be assessed by experts in the domain of application [23], something they are often reluctant to do because they do not feel familiar enough with the concept of probability or they find it difficult to attach a number to their beliefs [24]. This is exactly the problem we ran into, and none of the standard elicitation methods could help us overcome it. Indirect methods, such as lotteries, proved to be too complex and too time-consuming for eliciting the 4000 probabilities. Neither did our experts appreciate direct elicitation methods, such as a numerical scale with labelled anchors, although these are easy to understand and use and therefore less time-consuming. In this they agree with many others who, except in situations where the odds are objectively measurable, feel more at ease with verbal probability expressions than with numbers (e.g. [3,25-27]). For a detailed and comprehensive survey of the large body of literature on the subject of verbal probability expressions, see [28], or [29]. Since our primary interest was in fast and coherent elicitation, possibly later to be followed by more fine-grained assessments where necessary, we set out to design a probability scale with verbal labels as anchors to accommodate our experts, and numerical labels to inform the experts how their assessments would be translated to the point probabilities we required for the specification of our Bayesian network. In a later stage, we could use sensitivity analyses [30] to determine which assessments possibly needed refinement to ensure accurate behaviour of the final network.

#### Design of the scale

The design of the verbal-numerical probability scale is described in detail in [28]. Briefly: we conducted four studies to indirectly obtain a relation between verbal expressions and numerical interpretations of these expressions. Based on the results of these studies we constructed the scale shown in Figure 1a. It is a continuous scale, to allow subjects to indicate any degree of probability. In addition, the verbal probability labels are not placed in alignment with the numerical anchors, since the verbal expressions should not be taken to be in one-to-one correspondence with particular numbers, but rather as a set of labels with a stable rank-ordering, covering the whole probability continuum.

With this elicitation method, which included the verbal-numerical probability scale, the experts involved were able to give their assessments at a rate of 150 to 175 per hour. The experts indicated that they found the presence of both numerical and verbal labels next to the scale quite helpful. They had used words as well as numbers when thinking about their assessments, depending on how familiar they had felt with the situation to be assessed: the more uncertain they had felt, the more they had been inclined to think in verbal terms.

### This study

The aim of the current study was to extend our findings to a more realistic setting, with experts assessing probabilities for situations they encounter on a daily basis. We used three different probability response scales: 1) our 'double' verbal-numerical response scale; 2) a 'numerical' scale with numerical labels only, the labels being the same as the numerical labels on the double scale; and 3) a 'verbal' scale with only the verbal labels taken from the double scale (see Figure 1a, b and 1c). Using a within-subject design, we sought to provide an answer to the following questions: 1. which scale do GPs prefer?; 2. does the type of scale affect GPs' assessments and their confidence in these assessments?; and 3. does the type of scale influence unpacking effects?

The fact that people have been found to prefer the use of words to convey uncertainty was one of the reasons for us to include verbal labels on our probability response scale. As this observation also holds for physicians (see e.g. [3,26,27]) and is conform the observations from our first evaluation study, we predict that our subjects prefer the double scale to the numerical scale. We included a verbal scale in this study for the sake of completeness, but we expect that that particular scale gives the subjects too little to go by. For this same reason we predict that the assessments given on the verbal scale will differ from those given on the double scale or the numerical scale, but that the vagueness provided by the verbal scale will increase the subjects' confidence in their assessments. Given our previous experiences, we expect no difference in assessments between the double scale and the numerical scale, but do expect more confident assessments with the double scale. To the best of our knowledge, the studies that reveal the unpacking effect (e.g. [6,7,31]) have asked subjects to give probability assessments without providing any support. We expect that the use of a probability response scale as supporting tool will not take away the unpacking effect, but hopefully the effect will be decreased.

## Methods

### Participants

We purchased, from an institute for primary care, a list of 300 randomly selected addresses of Dutch practising General Practitioners (GPs). We sent these GPs a letter introducing our study and the request to participate, together with a questionnaire and a stamped return envelope. We did not offer any payment. We reassured the GPs that their answers would be analysed anonymously.

### Materials

#### Vignettes

We prepared 15 descriptions of common medical situations, each accompanied by probability questions concerning diagnostic, prognostic, or therapeutic alternatives. These vignettes were reviewed by two very experienced GPs (more than 20 years in practice), who judged their familiarity and plausibility.

The first three vignettes required the assessment of probabilities for alternative, mutually exclusive and exhaustive, diagnoses. We will refer to these as the multiple diagnoses vignettes. Each of these three multiple diagnoses vignettes had two versions, a short version and a long version (see Additional file 1, first three vignettes). The short version asked for the assessment of probabilities for two alternative diagnoses and the option 'other'; in the long, unpacked version, this 'other' option was replaced by three additional diagnoses plus the option 'other'. This manipulation is similar to the one used in fault tree studies in the area of analysing the fallibility of complex systems (cf. [32]), but has fixed options to study possible unpacking effects. Each vignette described a medical situation followed by the question: "Given that the patient has only one of the following illnesses, how likely do you think that illness is?", after which followed either the short or the long list of options. The remaining twelve vignettes each required the assessment of only a single probability (see Additional file 1, vignettes 4 through 15). We will refer to these as the simple vignettes. They concerned daily encountered medical situations together with probability assessment questions.

Each vignette displayed one of the three probability response scales for each probability to be assessed, that is, 3 or 6 scales for each of the multiple diagnoses vignettes and 1 scale for each simple vignette. Only one type of scale was used per vignette, the verbal, numerical or double type, depending on the version of the questionnaire.

#### Questionnaires

Each questionnaire started with the three multiple diagnoses vignettes, displaying a different type of response scale with each vignette. We rotated presentation order of the scales to counteract order effects, thus one third of the GPs assessed the first vignette on a verbal scale, one third started with a double scale and one third with the numerical scale. Observing that there are six possible combinations of the short and long versions of the three multiple diagnoses vignettes (excluding combinations of only short versions, or only long versions), we thus arrived at 18 different versions of the questionnaire for the first three vignettes. Each of these versions in addition contained the 12 simple vignettes, four with the verbal scale, four with the numerical scale and four with the double scale, again rotating presentation order in the different versions.

Immediately below the first vignette on page one of the questionnaire, we presented the statement "This scale was very usable for indicating my assessment"; with yes, undecided or no as response options. This question enabled us to establish a primary reaction to usefulness of the scale, not influenced by having seen the other scales. Moreover, since different groups of GPs were presented with different scales first, we were able to compare these initial evaluations between subjects.

For each of the 15 vignettes we included a question about the GP's confidence in his/her probability assessment(s). Confidence was to be indicated on a horizontal line with complete/100% at one end and no/0% at the other.

On the final page of the questionnaire we printed the question: "If you were asked to assess another 500 situations similar to the ones you just assessed, which scale would you prefer to use?", where participants could tick verbal, verbal plus numerical, or numerical. We left room for remarks, and asked for their gender, year of birth and years of practice as GP.

#### Data preparation

All probability and confidence assessments were measured with a ruler, anchored at 0 at the lowest and 100 at the highest point. For the three multiple diagnoses vignettes, the short lists (packed version) each contain two alternative diagnoses, and the option 'other'; we will denote these options by A, B, and O. In the long lists, the first two diagnoses were exactly the same as the first two in the short lists: A and B. The four remaining options in the long lists should therefore together be equally probable as the option 'other' of the short lists. For our analyses, we sum the probability assessments for these latter four options from the long lists and take that sum as the assessment for the compound option O in the unpacked version.

## Results

### Participants

Eight questionnaires were returned uncompleted, either because the addressee had moved (4) or because the GP did not have the time or motivation to participate (4). After four weeks and a reminder, we had received 86 completed questionnaires: a response rate of 29%. For the returned questionnaires we found close to equal numbers of respondents per version.

Of the 86 GPs who responded, 27 were women, 57 were men and two GPs had not given their gender. The women had a mean age of 47 years (SD = 7.3) and the men of 50 years (SD = 6.9). The men had a mean of 19 years (SD = 8.4) of practice as a GP, and the women 15 years (SD = 7.4).

### Usability and preference

As indicated on the first page of the questionnaire, i.e. after they had assessed one multiple diagnoses vignette with one of the three scales, every participant thought their scale was quite usable, whether it was the verbal scale, the double, or the numerical scale (70, 73 and 74% 'yes' answers to the usability question for the three scales, respectively). We checked whether this appreciation of the scale presented first biased participants in their ultimate preference: would participants who had started out with one scale like that scale most in the end, as indicated on the last page in answer to the question which scale they would prefer to use if they were asked to assess another 500 situations? Table 1 shows that this was not the case (χ^2^(6) = 9.511, p = .147). Thus the first scale used did not bias preferences. Table 1 also shows that there was no general preference for one of the three scales as indicated on this final question: the verbal scale was preferred by 20 participants, 26 participants preferred the double scale and 34 participants the numerical scale. These preferences did not differ significantly (χ^2^(2) = 3.7, p = .157). Gender did not affect preference either (χ^2^(2) = .965, p = .617).

**Table 1:** First and preferred scale: Number of participants with the first scale used and their preferred scale

	Preference				
			
first used	verbal	double	numerical	missing	total
Verbal	6	15	11	1	33
Double	7	4	11	4	26
numerical	7	7	12	1	27
Total	20	26	34	6	86

We did find differences when we took years of experience of the participant into account. We divided the GPs in four almost equally sized experience-groups. As shown in Table 2, the differences in preference were significant (χ^2^(6) = 14.856, p = .021). The least experienced group preferred words, the middle groups preferred the double scale, and the most experienced GPs preferred numbers.

**Table 2:** Experience and preference: Numbers of participants per experience-group who stated a preference (n = 80), and their preferred scale

	Preference			
		
years of experience	verbal	double	numerical	total
2–11	11	5	6	22
12–18	5	10	9	24
19–25	0	7	10	17
26–30	4	4	9	17
Total	20	26	34	80

### Probability and confidence assessments

We analysed the relations between probability assessments and scale type for the twelve simple vignettes. We found that for none of the twelve vignettes did the type of scale used result in significantly different assessments, see Table 3. Neither was there a trend for assessments to be consistently higher or lower on one scale than on the other scales.

**Table 3:** Probability and confidence: Mean probability and confidence assessments (plus standard deviations) for the simple vignettes, per scale type

	probability			Confidence		
	
Vignette	verbal	double	numerical	verbal	double	numerical
4	69 (16.7)	65 (20.0)	74 (18.3)	82 (12.2)	83 (12.5)	81 (17.2)
5	40 (22.5)	47 (22.3)	41 (23.2)	63 (22.9)	73 (21.5)	68 (21.1)
6	58 (27.2)	69 (22.3)	66 (18.2)	83 (11.5)	80 (14.9)	72 (21.0)
7	42 (21.6)	42 (17.4)	44 (21.5)	78 (13.4)	77 (17.2)	76 (18.7)
8	54 (22.1)	44 (22.0)	41 (21.4)	80 (16.9)	74 (24.1)	76 (18.1)
9	15 (7.0)	17 (10.5)	14 (12.2)	89 (5.8)	86 (14.2)	89 (7.5)
10	32 (19.6)	35 (19.0)	34 (20.4)	82 (14.8)	77 (14.6)	77 (16.0)
11	49 (19.3)	46 (21.0)	41 (20.8)	76 (18.5)	76 (15.4)	72 (19.4)
12	40 (17.5)	43 (21.5)	40 (21.8)	81 (12.9)	82 (15.0)	76 (15.4)
13	80 (24.2)	72 (23.9)	76 (24.8)	88 (14.2)	80 (24.4)	86 (14.4)
14	90 (16.9)	94 (7.2)	85 (22.4)	92 (10.7)	93 (9.4)	92 (8.0)
15	57 (33.1)	62 (28.7)	76 (18.9)	87 (12.1)	88 (12.5)	89 (11.6)

For the simple vignettes, confidence was generally quite high, ranging from a mean of 72 to 89, with SDs between 12 and 20, see Table 3. They were nowhere significantly different from each other for the same vignette with the different scales.

Agreement among GPs both in their probability ratings and in their confidence ratings for the twelve vignettes was significant, but not impressive: Kendall's coefficient for concordance had the value W = .52 for the probability ratings, and only W = .24 for the confidence ratings (in both cases df = 11, p < .00).

We found that neither age nor experience nor gender was related to the probability assessments or to the confidence assessments with any of the three types of scale.

### Subadditivity and unpacking effects

With the three multiple diagnoses vignettes, participants had to assess probabilities for an entire distribution of mutually exclusive and exhaustive hypotheses, that is, per vignette the assessed probabilities should sum to 100%. In addition, the assessments for the options A, B and O in the short, packed, versions should be equal to the respective assessments for the options A, B and the compound O in the long, unpacked, version. There is subadditivity if the estimates for options A, B and O add up to more than 100%. There is an unpacking effect if the probability assessment for the compound option O in the long list is higher than that for the O option in the short list.

We see subadditivity with all three multiple diagnoses vignettes, and both with a short and with a long list of options, regardless of the response scale used. For each vignette a 2 (List: long or short) × 3 (Scale: verbal, double or numerical) × 3 (Option A, B or O) ANOVA was performed, with list and scale as between subjects factors and option as within subject factor. These analyses showed that with all vignettes there were significant differences depending on whether the list was long or short (vignette 1: F(1,79) = 27.908, p = .000; vignette 2: F(1,78) = 39.272, p = .000; vignette 3: F(1,78) = 48.316, p = .000). Thus subadditivity was significantly more apparent after unpacking. We observe from Table 4 that the average assessments given for the A and B options are comparable per response scale and vignette. The difference in subadditivity between the short and long versions is therefore purely due to unpacking effects, resulting in a more substantial overestimation of the probabilities assessed for the O option in the long list than in the short list.

**Table 4:** Mean probability assessments (plus standard deviations) for multiple diagnoses vignettes 1–3

		vignette 1		vignette 2		vignette 3	
		
option	scale	short list (n = 32)	long list (n = 54)	short list (n = 62)	long list (n = 24)	short list (n = 42)	long list (n = 44)
	verbal	18 (4.8)	31 (21.6)	10 (8.6)	9 (8.1)	59 (26.7)	50 (24.9)
A	double	24 (21.5)	30 (14.7)	9 (6.6)	7 (5.6)	59 (23.6)	66 (20.2)
	numerical	29 (22.7)	28 (21.0)	8 (4.6)	4 (0.9)	65 (26.9)	61 (25.2)
	total	25 (19.9)	30 (19.3)	9 (6.8)	6 (5.2)	62 (25.5)	58 (24.0)
	verbal	36 (20.4)	28 (15.3)	82 (13.9)	89 (13.8)	27 (10.3)	28 (16.0)
B	double	28 (15.6)	32 (18.1)	75 (29.4)	72 (23.1)	19 (14.4)	29 (16.9)
	numerical	30 (22.4)	26 (20.4)	81 (9.6)	80 (10.7)	21 (14.5)	26 (15.1)
	total	31 (20.1)	29 (17.4)	79 (21.1)	78 (18.8)	22 (13.4)	28 (15.8)
	verbal	63 (22.4)	121 (36.3)	32 (17.7)	96 (25.3)	46 (29.3)	128 (50.4)
O	double	76 (15.2)	130 (39.0)	37 (26.8)	85 (31.4)	39 (23.7)	108 (38.9)
	numerical	63 (12.9)	117 (53.5)	28 (26.6)	74 (66.0)	30 (24.6)	88 (60.2)
	total	66 (16.3)	123 (41.7)	33 (24.2)	84 (43.2)	37 (26.1)	112 (50.6)

In addition, we found for only vignette 3 that the extent of subadditivity due to unpacking significantly depended on the scale used (F(2,78) = 2.84, p = .045). It was strongest with the verbal scale and weakest for the numerical scale, the double scale falling in between. For the other two vignettes there was no interaction effect of scale and option.

## Discussion

### Which scale do GPs prefer?

The answer to this first research question is that altogether the GPs in our study were not partial to any scale. We did find that less experienced GPs preferred words, and more experienced GPs favoured numbers. This is in line with the observations made by the experts whose probability assessments we elicited for the oesophagus network (see above), that people who are less knowledgeable about (part of) a domain and thus more uncertain, prefer to use words to express this uncertainty, rather than numbers with their seemingly precise meaning.

### Does the type of scale affect GPs' assessments and confidence?

We found that the type of scale used did not seem to affect the probability and confidence assessments. Confidence was generally found to be quite high. Although this study was not designed to test accuracy of assessments, the agreement between GPs in probability assessments over the three scales, suggests that the type of scale used will not affect the accuracy. This is in line with our previous findings (see [17]).

### Does the type of scale influence unpacking effects?

As all our multiple diagnoses vignettes included more than two hypotheses, it is not surprising that subadditivity was found to be a general phenomenon [6]. The extent of additional subadditivity due to unpacking did not, in general, seem to depend on the scale used. From our analyses for vignette 3, however, we conclude that use of the verbal response scale involves a risk of significantly more overestimation upon unpacking.

We can compare our results for vignette 1 to those given by Redelmeier et al's subjects (in [6]) to this same vignette. Our subjects overestimated the option O in the long list much more (mean of 123) than Redelmeier's (mean of 69; see [6]). Although their subjects still showed unpacking effects (option O's assessment increased from 50 in the packed version to 69 in the unpacked version), possibly Redelmeier's exhortation to make sure that the estimates add up to no more than 100% was stronger than our implicit statement that the options were mutually exclusive and exhaustive: "Given that the patient has only one of the following illnesses...". Indeed, if we rescale the assessments we found to a 0%–100% scale, we find the same mean assessments as Redelmeier et al.

Although our results show that in general it does not matter which of the three response scales is used whenever a probability response scale is chosen as supporting tool, remarks made indicate that individual preferences do exist. Subjects who preferred the verbal scale, said for example: "I feel more at ease with words; I don't really work with numerical assessments myself", and "I can't do much with numbers; words are much more meaningful to me". Also: "I feel the numbers force me to give a more precise answer, it's more gradual with words." Others, who preferred the numerical scale, remarked for example: "I thought the words were disturbing.", "I would even prefer a blank line."

Those with a preference for the numerical scale did see the advantage of words, though: "As an introduction, it is nicer to see words too, after that I don't look at them anymore", as well as the disadvantages: "Words carry with them a stronger suggestion that something is almost impossible, while in practice you do have to take the possibility into account. I found I tended to go higher on the scale." These and similar thoughts about the consequences made them prefer numbers: "Words are more exact for me, but take longer and are more difficult to process, I think." It is interesting that another GP, who also preferred numbers, said that "Numbers are more exact and less dependent on subjective interpretation." So words are more exact to some, and numbers are more exact to others.

## Conclusion

We conclude that the different types of probability scale are equally suitable for supporting probability assessments. In addition, we advise that to counter subadditivity subjects should estimate the whole distribution of options together, and it should be enforced that the estimates add up to exactly 100%. To diminish unpacking effects, options should describe well-defined events and nothing vague like 'other'. We finally advise that the verbal-numerical probability scale is a good option for aiding probability assessment: it offers numerical labels to those who prefer numbers and verbal labels to those who prefer words, thus accommodating both more and less experienced professionals in both more and less uncertain situations. The double scale may also serve well in the communication of probabilistic information and risks, e.g. by doctors to patients. Since people differ in their preferences for verbal or numerical terms but are willing to use both [33], the double scale might be the tool to give an on-the-spot translation of words into numbers and vice versa.

## Competing interests

The author(s) declare that they have no competing interests.

## Authors' contributions

CLMW and SR conceived of and carried out the study, and drafted the manuscript. PK participated in the design of the study, performed the statistical analyses and took care of the final formatting of the manuscript. All authors read and approved the final manuscript.

## Pre-publication history

The pre-publication history for this paper can be accessed here:



## Supplementary Material

Additional File 1 The vignettes. Click here for file

## References

[B1] Pauker SG, Kassirer JP (1980). The threshold approach to clinical decision making. New England Journal of Medicine.

[B2] Tversky A, Kahneman D (1974). Judgment under Uncertainty: Heuristics and Biases. Science.

[B3] Kuipers B, Moskowitz AJ, Kassirer JP (1988). Critical decisions under uncertainty: representation and structure. Cognitive Science.

[B4] Timmermans D, Kievit J, Van Bockel H (1996). How do surgeons' probability estimates of operative mortality compare with a decision analytic model?. Acta Psychologica.

[B5] Tversky A, Koehler DJ (1994). Support Theory: A nonextensional representation of subjective probability. Psychological Review.

[B6] Redelmeier DA, Koehler DJ, Liberman V, Tversky A (1995). Probability judgment in medicine: Discounting unspecified possibilities. Medical Decision Making.

[B7] Cahan A, Gilon D, Manor O, Paltiel O (2003). Probabilistic reasoning and clinical decision-making: do doctors overestimate diagnostic probabilities?. QJM: An International Journal of Medicine.

[B8] Spetzler CS, Stael von Holstein CAS (1975). Probability encoding in decision analysis. Management Science.

[B9] Renooij S (2001). Probability elicitation for belief networks: Issues to consider. Knowledge Engineering Review.

[B10] O'Hagan A, Buck CE, Daneshkhah A, Eiser JR, Garthwaite PH, Jenkinson DJ, Oakley JE, Rakow T (2006). Uncertain Judgements: Eliciting experts' probabilities.

[B11] Van der Gaag LC, Renooij S, Witteman CLM, Aleman BMP, Taal BG (2002). Probabilities for a probabilistic network: A case study in oesophageal cancer. Artificial Intelligence in Medicine.

[B12] Woloshin S, Schwartz LM, Byram S, Fischhoff B, Welch G (2000). A new scale for assessing perceptions of chance: A validation study. Medical Decision Making.

[B13] Charitos Th, Van der Gaag LC, Visscher S, Schurink K, Lucas PJF, Holmes JH, Peek NB (2005). A dynamic Bayesian network for diagnosing ventilator-associated pneumonia in ICU patients. Proceedings of the 10th Intelligent Data Analysis in Medicine and Pharmacology Workshop.

[B14] Vicari RM, Flores CD, Silvestre AM, Seixas LJ, Ladeira M, Coelho H (2003). A multi-agent intelligent environment for medical knowledge. Artificial Intelligence in Medicine.

[B15] Geenen PL, Van der Gaag LC (2005). Developing a Bayesian network for clinical diagnosis in veterinary medicine: from the individual to the herd. Proceedings of the Third Bayesian Modelling Applications Workshop; Edinburgh.

[B16] Populaire S, Ginestet P, Blanc J, Denoeux T (2002). Fusion of expert knowledge with data using belief functions: a case study in waste-water treatment. Proceedings of the Fifth International Conference on Information Fusion.

[B17] Witteman CLM, Renooij S (2003). Evaluation of a verbal-numerical probability scale. International Journal of Approximate Reasoning.

[B18] Pearl J (1988). Probabilistic Reasoning in Intelligent Systems: Networks of Plausible Inference.

[B19] Andreassen S, Woldbye M, Falck B, Andersen SK (1987). MUNIN. A causal probabilistic network for interpretation of electromyographic findings. Proceedings of the 10th International Joint Conference on AI.

[B20] Korver M, Lucas PJF (1993). Converting a rule-based expert system into a belief network. Medical Informatics.

[B21] Dietz FJ, Mira J, Iturralde E, Zubillage Z (1997). DIAVAL, a Bayesian expert system for echocardiography. AI in Medicine.

[B22] Lucas PJF, Boot H, Taal BG (1998). Computer-based decision-support in the management of primary gastric non-Hodgkin lymphoma. Methods of Information in Medicine.

[B23] Druzdzel MJ, Van der Gaag LC (2000). Building probabilistic networks: where do the numbers come from?. IEEE Transactions in Knowledge and Data Engineering.

[B24] Henrion M, Pradhan M, Del Favero B, Huang K, Provan G, O'Rorke P (1996). Why is diagnosis using belief networks insensitive to imprecision in probabilities?. Proceedings of the 12th Conference on Uncertainty in AI.

[B25] Brun W, Teigen KH (1988). Verbal probabilities: ambiguous, context-dependent, or both?. Organizational Behavior and Human Decision Processes.

[B26] Merz JF, Druzdzel MJ, Mazur DJ (1991). Verbal expressions of probability in informed consent litigation. Medical Decision Making.

[B27] Erev I, Cohen BL (1990). Verbal versus numerical probabilities: efficiency, biases, and the preference paradox. Organizational Behavior and Human Decision Processes.

[B28] Renooij S, Witteman CLM (1999). Talking probabilities: communicating probabilistic information with words and numbers. International Journal of Approximate Reasoning.

[B29] Teigen KH, Brun W, Hardman D, Macchi L (2003). Verbal expressions of uncertainty and probability. Thinking: Psychological perspectives on reasoning, judgment and decision making.

[B30] Coupé VMH, Van der Gaag LC, Habbema JDF (2000). Sensitivity analysis: an aid for probability elicitation. Knowledge Engineering Review.

[B31] Sloman S, Rottenstreich Y, Wisniewski E, Hadjichristidis C, Fox CR (2004). Typical versus atypical unpacking and superadditive probability judgment. Journal of Experimental Psychology: Learning, Memory and Cognition.

[B32] Fischhoff B, Slovic P, Lichtenstein S (1978). Fault trees: Sensitivity of estimated failure probabilities to problem representation. Journal of Experimental Psychology: Human Perception and Performance.

[B33] Wallsten TS, Budescu DV, Zwick R, Kemp SM (1993). Preferences and reasons for communicating probabilistic information in verbal or numerical terms. Bulletin of the Psychonomic Society.

[B34] Wallsten TS, Fillenbaum S, Cox JA (1986). Base rate effects on the interpretations of probability and frequency expressions. Journal of Memory and Language.

