# Supplementary material for: Medicine in words and numbers: a cross-sectional survey comparing probability assessment scales
Source: BMC Med Inform Decis Mak. 2007 Jun 11;7:13. doi: 10.1186/1472-6947-7-13 (PMC1903351; doi:10.1186/1472-6947-7-13)
Supplement: Additional File 1 — The vignettes. [file 1472-6947-7-13-S1.doc]

The vignettes

1. A well-known 22-year-old actress presents with pain in the right lower quadrant of her abdomen of 12 hours' duration. Her last normal menstrual period was 4 weeks ago. Given that she has only one illness, how likely do you think that illness is:

+ short option list: gastroenteritis, ectopic pregnancy, other

+ long option list: gastroenteritis, ectopic pregnancy, appendicitis, pyelonephritis, pelvic inflammatory disease, other

(taken from [6])

2. A 24-year old student has had, for some days now, intermittent pain in the left chest area, next to the sternum. Given that he has only one illness, how likely do you think that illness is:

+ short option list: angina pectoris, atypical thoracic pain, other

+ long option list: angina pectoris, atypical thoracic pain, infarct, hyperventilation, lung emboly, other

3. A 38-year old woman, not using oral or spiral contraceptives, suffers from vaginal bleeding between periods. Given that she has only one illness, how likely do you think that illness is:

+ short option list: fluctuation in hormone levels, myoma, other

+ long option list: fluctuation in hormone levels, myoma, cervical polyp, STD, cervical carcinoma, other

4. A 30-year old computer programmer at a large software house presents with stomach problems. He drinks 10-12 cups of strong coffee a day. How likely do you think that his stomach problems will cease to exist if he stops drinking coffee? (adapted from [34])

5. A 28-year old secretary at a large bank presents with headaches. She smokes 30-40 cigarettes a day. How likely do you think that her headaches will disappear if she stops smoking?

6. A 32-year old academic researcher presents with concentration problems. For the last year, she hasn't slept a lot at night. How likely do you think that her concentration will improve if she (without medication) would sleep better?

7. You remove a wart from a patient's hand. How probable do you think it is that the wart grows back within three months? (adapted from [34])

8. You treat a calcified toenail. How probable do you think it is that the calcification returns within a year?

9. You vaccinate a healthy 65-year old woman against the flu. How likely do you think it is that she will suffer from side-effects? (adapted from [34])

10. A 45-year old civil servant severely complains about radiating lower back pain. This has been going on for the last 3 weeks. How likely do you think it is, before investigating any further, that he has a hernia?

11. A 10-year old child complains repeatedly about headaches. Before investigating any further, how probable do you think it is that the child needs glasses?

12. A 16-year old girl repeatedly complains about hearing loss. Before investigating any further, how likely do you think it is that she requires ear syringing?

13. Your 56-year old patient calls with complaints about chest pain. How likely is it that you will visit him?

14. A 63-year old woman had one-time postmenopausal blood loss. How probable do you think it is that you will perform examinations additional to physical examination?

15. A patient has twisted his ankle during a soccer match. The ankle is severely swollen and the patient is unable to put any weight on the injured foot. How high is the probability that you will decide to order an X-ray? (adapted from [34])
